# Supplementary material for: The Utility of Three-Dimensional Printing in Physician-Modified Stent Grafts for Aortic Lesions Repair
Source: J Clin Med. 2024 May 18;13(10):2977. doi: 10.3390/jcm13102977 (PMC11122058; doi:10.3390/jcm13102977)
Supplement: Supplementary file 1 [file jcm-13-02977-s001.zip › jcm-2979717-supplementary.pdf]

PRISMA 2020 Checklist

Supplementary File S1 Prisma 2020 Checklist

Section and Topic	Item #	Checklist item	Location where item is reported
**TITLE**			
Title	1	Identify the report as a systematic review.	1,2
**ABSTRACT**			
Abstract	2	See the PRISMA 2020 for Abstracts checklist.	20,105
**INTRODUCTION**			
Rationale	3	Describe the rationale for the review in the context of existing knowledge.	31-92
Objectives	4	Provide an explicit statement of the objective(s) or question(s) the review addresses.	93-96
**METHODS**			
Eligibility criteria	5	Specify the inclusion and exclusion criteria for the review and how studies were grouped for the syntheses.	107-129
Information sources	6	Specify all databases, registers, websites, organisations, reference lists and other sources searched or consulted to identify studies. Specify the date when each source was last searched or consulted.	131-144
Search strategy	7	Present the full search strategies for all databases, registers and websites, including any filters and limits used.	139
Selection process	8	Specify the methods used to decide whether a study met the inclusion criteria of the review, including how many reviewers screened each record and each report retrieved, whether they worked independently, and if applicable, details of automation tools used in the process.	145-161
Data collection process	9	Specify the methods used to collect data from reports, including how many reviewers collected data from each report, whether they worked independently, any processes for obtaining or confirming data from study investigators, and if applicable, details of automation tools used in the process.	145-161
Data items	10a	List and define all outcomes for which data were sought. Specify whether all results that were compatible with each outcome domain in each study were sought (e.g. for all measures, time points, analyses), and if not, the methods used to decide which results to collect.	164-186
	10b	List and define all other variables for which data were sought (e.g. participant and intervention characteristics, funding sources). Describe any assumptions made about any missing or unclear information.	173-186, 199-201
Study risk of bias assessment	11	Specify the methods used to assess risk of bias in the included studies, including details of the tool(s) used, how many reviewers assessed each study and whether they worked independently, and if applicable, details of automation tools used in the process.	189-193
Effect measures	12	Specify for each outcome the effect measure(s) (e.g. risk ratio, mean difference) used in the synthesis or presentation of results.	146-209
Synthesis methods	13a	Describe the processes used to decide which studies were eligible for each synthesis (e.g. tabulating the study intervention characteristics and comparing against the planned groups for each synthesis (item #5)).	237-250
	13b	Describe any methods required to prepare the data for presentation or synthesis, such as handling of missing summary statistics, or data conversions.	199-201
	13c	Describe any methods used to tabulate or visually display results of individual studies and syntheses.	237-250, 275-290
	13d	Describe any methods used to synthesize results and provide a rationale for the choice(s). If meta-analysis was performed, describe the model(s), method(s) to identify the presence and extent of statistical heterogeneity, and software package(s) used.	258-269
	13e	Describe any methods used to explore possible causes of heterogeneity among study results (e.g. subgroup analysis, meta-regression).	280-287
	13f	Describe any sensitivity analyses conducted to assess robustness of the synthesized results.	-
Reporting bias assessment	14	Describe any methods used to assess risk of bias due to missing results in a synthesis (arising from reporting biases).	291-318

PRISMA 2020 Checklist

Section and Topic	Item #	Checklist item	Location where item is reported
Certainty assessment	15	Describe any methods used to assess certainty (or confidence) in the body of evidence for an outcome.	275-317
**RESULTS**			
Study selection	16a	Describe the results of the search and selection process, from the number of records identified in the search to the number of studies included in the review, ideally using a flow diagram.	162,163
	16b	Cite studies that might appear to meet the inclusion criteria, but which were excluded, and explain why they were excluded.	159-161
Study characteristics	17	Cite each included study and present its characteristics.	237-250
Risk of bias in studies	18	Present assessments of risk of bias for each included study.	291-318
Results of individual studies	19	For all outcomes, present, for each study: (a) summary statistics for each group (where appropriate) and (b) an effect estimate and its precision (e.g. confidence/credible interval), ideally using structured tables or plots.	237-250
Results of syntheses	20a	For each synthesis, briefly summarise the characteristics and risk of bias among contributing studies.	291-318
	20b	Present results of all statistical syntheses conducted. If meta-analysis was done, present for each the summary estimate and its precision (e.g. confidence/credible interval) and measures of statistical heterogeneity. If comparing groups, describe the direction of the effect.	260-269
	20c	Present results of all investigations of possible causes of heterogeneity among study results.	280-284
	20d	Present results of all sensitivity analyses conducted to assess the robustness of the synthesized results.	280-285
Reporting biases	21	Present assessments of risk of bias due to missing results (arising from reporting biases) for each synthesis assessed.	291-318
Certainty of evidence	22	Present assessments of certainty (or confidence) in the body of evidence for each outcome assessed.	280-285
**DISCUSSION**			
Discussion	23a	Provide a general interpretation of the results in the context of other evidence.	320-487
	23b	Discuss any limitations of the evidence included in the review.	489-505
	23c	Discuss any limitations of the review processes used.	489-505
	23d	Discuss implications of the results for practice, policy, and future research.	507-515
**OTHER INFORMATION**			
Registration and protocol	24a	Provide registration information for the review, including register name and registration number, or state that the review was not registered.	106
	24b	Indicate where the review protocol can be accessed, or state that a protocol was not prepared.	139
	24c	Describe and explain any amendments to information provided at registration or in the protocol.	101-196
Support	25	Describe sources of financial or non-financial support for the review, and the role of the funders or sponsors in the review.	522
Competing interests	26	Declare any competing interests of review authors.	532
Availability of data, code and other materials	27	Report which of the following are publicly available and where they can be found: template data collection forms; data extracted from included studies; data used for all analyses; analytic code; any other materials used in the review.	525

PRISMA 2020 Checklist

From: Page MJ, McKenzie JE, Bossuyt PM, Boutron I, Hoffmann TC, Mulrow CD, et al. The PRISMA 2020 statement: an updated guideline for reporting systematic reviews. BMJ 2021;372:n71. doi: 10.1136/bmj.n71

For more information, visit: http://www.prisma-statement.org/

Supplementary File S2 Systematic Review Search Strategy

Systematic Review Search Strategy

Review title: The Role of Three-Dimensional Printing in Approaches in Endovascular Aortic Aneurysm Repair

Review team: Wiktoria Zasada, Magdalena Węglewska, Jerzy Kluba, Łukasz Świątek, Hubert Stępak, MD, PhD, prof. Zbigniew Krasiński MD, PhD

The search strategy was developed by and will be conducted by Wiktoria Zasada. The strategy was peer-reviewed and piloted and will be updated as needed based on database-specific language or other requirements within the following databases:

• MEDLINE (PubMed)
• CAB Abstracts (Web of Science)
• CINAHL (Ebsco)
• Web of Science Core Collection (Web of Science)
• EMBASE
• Cochrane Library
• ClinicalTrials.gov
• Scopus Science Direct
• Google Scholaropengrey.eu/

For non-indexed conference proceedings, the review team will search relevant conference proceedings and websites (e.g. ProceedingsFirst, CDC, Google Scholar, OpenGrey.eu). In addition, the review team will hand-search bibliographies of relevant systematic reviews, narrative reviews, and meta-analyses found, as well as and relevant citations bibliographies of the articles included in the review.

We will not include or exclude studies based on the publication dates, and only content published or available in English will be included. To ensure the content accurately reflects research reported within the review's proposed time frame, monthly search alerts will be established for each database and monitored after the initial search, and eligible articles will be added to our review through the data extraction phase

Supplementary File S2 Systematic Review Search Strategy

Version of a systematic review search strategy constructed for MEDLINE (PubMed).

#	Search strategy	# of results
1	Aortic Aneurysm[mh] OR Aortic Aneurysm, Abdominal[mh] OR Aortic Aneurysm, Thoracic[mh] OR Aortic Aneurysm, Ruptured[mh] OR Abdominal Aortic Aneurysm[tiab] OR Thoracic Aortic Aneurysm[tiab] OR Aneurysm, False[tiab] OR AAA[tiab] OR AAAs[tiab] OR Endovascular Aortic Repair[tiab] OR Aortic Stent Grafting[tiab] OR Fenestrated Endovascular Aneurysm Repair[tiab] OR FEVAR[tiab] OR Aortic Aneurysm Repair[tiab] OR Juxtarenal Aneurysm[tiab] OR Physician Modified[tiab] OR Surgeon Modified[tiab] OR Surgeon-Modified[tiab] OR Surgeon-Modified Stent Graft[tiab] OR Surgeon Modified Stent Graft[tiab] OR Physician-Modified Stent Graft[tiab] OR Physician Modified Stent Graft[tiab] OR PMSGs[tiab] OR PMSG[tiab] OR Template-Assisted Stent Graft[tiab]	78,887
2	Three-Dimensional Printing[mh] OR 3D Printing[mh] OR Three-Dimensional Print*[tiab] OR 3D Print*[tiab] OR 3-D Print*[tiab] OR 3d-printing[tiab] OR Patient-Specific Modeling[tiab] OR Personalized Printing[tiab] OR Template-Assisted Printing[tiab] OR (three dimensional printing OR three-dimensional printing OR 3D printing OR 3d printing OR 3-d printing OR 3d-printing OR patient-specific modeling OR personalized printing OR template-assisted printing)	49,452
3	#1 AND #2	270

Supplementary File S3 Table of studies and extracted variables

	Author	Reference	Year of public	Country	Numbe r of patient	Type of surgery	Software	Model of the 3D printer	Polymer used for printing	Estim ated cost	Time spent for printing	Mean stent modification time	Name of the endograft modified	Sterilization technique	Mean time of cannulat	Fluoroscopy time	Contrast agent volume	Mean procedure time	Optima l angiogr	Average intraoperative blood loss	Mean hospital stay duration	Mean postoperati ve	30 day surviva l rate	Mean follow-up	Types of settings
Unit	-	-	-	-	-	-	-	-	-	USD	minutes	minutes	-	-	minutes	minutes	mL	minutes	%	mL	days	days	%	months	-
	Fu et al. [x]	cbi.nlm.ni	2023	China	44	FEVAR, BEVAR	Mimics, Geomagic Studio 2014, Geomagic Design Direct	Eden260VS	NA	NA	180	44.05 ± 7.72	Ankura, Valiant Captivia, Endurant, Fluency, Viabahn	Ethylene Oxide	NA	NA	134.59 ± 24.24	298.2 ± 84	100	480.91 (100–2810)	9.91 ± 4.47	1.02 (0-5)	100	6 for 42 patients, 12 for 35 patients	Experianced university center
	Rynio et al. [x]	cbi.nlm.nil	2022	Poland	43	FEVAR, BEVAR	3D Slicer 11.0, PreForm	Form 2	Standard clear resin	5 ± 2	361 ± 114	86 ± 12	Valiant Captiva	Hydrogen Peroxide plasma, Ethylene Oxide gas	NA	NA	17.67 ±36.70	247 ± 70	86.05	NA	8 ± 12	NA	88	14 ± 12	Center with no prior experience in complex endovascular aortic repairs
	Branzan et al. [x]	icle/S1078	2021	Germany	19	FEVAR	Geomagic DesignX 2019	Form 2	Biocompatible Dental SG resin	NA	420 (segmentation, printing, and post-processing)	109.6 ± 10.7	Valiant Captivia, Endurant	Steam pressure	78	55	77.7 ± 34.9	161±95	100	NA	17.3	2.8	100	14.4	The single centre, single surgeon experience from few years of performing these surgeries.
	Zheng et al. [x]	cbi.nlm.nil	2023	China	32	TEVAR	Mimics, Geomagic Studio 2014	Eden260VS	Photosensitive resin	410	NA	37.63 ± 2.99	Ankura	Ethylene Oxide	NA	NA	NA	147.84 ± 33.94	100	NA	NA	NA	100	16.14 ± 3.76	NA
	Tong et al. [x]	cbi.nlm.ni	2020	China	34	TEVAR	Mimics, Geomagic Studio 2014, EndoSize, CAD	Eden260VS	MED610 (Stratasys) materials	NA	180	75.6 ± 21	Ankura, Endurant, Zenith, Viabahn	Ethylene Oxide	NA	NA	224.58 ± 45.33	336 ± 72	100	355.48 ± 172.38	10.22 ± 3.65	0.82 (0–4)	100	8.5	Tertiary center with extensive clinical experience.
	hee et al. [	cbi.nlm.nil	2021	South Korea	20	TEVAR	NA	ProJet CJP	VisiJet PXL Core powder, VisiJet PXL clear binder, Color bonds	NA	NA	NA	Hemashield Platinum straight graft (MAQUET Cardiovascular LLC, San Jose, CA)	NA	NA	NA	NA	441 (IQR, 392.8-492.3)	100	500 (IQR, 300-800)	22 (IQR, 15-29)	6 (IQR, 5-10)	100	median: 35 (range 1-56 months) - data for joined group - with and without 3D printing	Repairs performed by a single surgeon in a high-volume center.

Supplementary File S4 The extracted variables with chosen units

Variable	Unit
Reference	N/A
Source	N/A
Country	N/A
Number of patients	N/A
Type of surgery	N/A
Software	N/A
Model of the 3D printer	N/A
Polymer used for printing	N/A
Estimated cost of printing	USD
Time spent for printing	minutes
Mean stent modification time	minutes
Name of the endograft modified	N/A
Sterilization technique	N/A
Mean time of cannulation	minutes
Fluoroscopy time	minutes
Contrast agent volume	milliliters
Mean procedure time	minutes
Optimal angiographic result obtained	%
Average intraoperative blood loss	milliliters
Mean hospital stay duration	days
Complications	N/A
Mean postoperative intensive care unit monitoring duration	days
30-day survival rate	%
Mean follow-up	months
Types of settings	N/A

Table 1. Extracted variables along with their respective units.

METHODOLOGICAL INDEX FOR NON-RANDOMIZED STUDIES (MINORS)

For: The Utility of Three-Dimensional Printing in Physician-Modified Stent-Grafts for Aortic Lesions Repair

Assessed study: Branzan et al. The Influence of 3D Printed Aortic Models on the Evolution of Physician Modified Stent Grafts for the Urgent Treatment of Thoraco-abdominal and Pararenal Aortic Pathologies

**1. A clearly stated aim**: the question addressed should be precise and relevant in the light of available literature	Score (0-2)
Answer: The aim was stated and relevant to available literature: “The aim was to describe the outcomes of high risk patients with symptomatic or contained rupture of pararenal (PRAs) and thoraco-abdominal aortic aneurysms (TAAAs) with anatomy unsuitable for commercially available stent grafts who underwent fenestrated endovascular aneurysm repair (FEVAR) using physician modified stent grafts (PMSGs) planned with 3D image analysis software (3DIMAS), and 3D printed aortic models (3DAMs).”	2
**2. Inclusion of consecutive patients**: all patients potentially fit for inclusion (satisfying the criteria for inclusion) have been included in the study during the study period (no exclusion or details about the reasons for exclusion)	
Answer: “Candidates for treatment with a PMSG were patients at anatomical and/or medical high risk of open repair,9 presenting with painful aneurysms, haemodynamically stable contained aortic ruptures, or symptomatic suture aneurysm after open AAA repair, where the available off the shelf branched stent grafts were not suitable due to anatomical constraints.”	2
3. **Prospective collection of data:** data were collected according to a protocol established before the beginning of the study	
Answer: It is an retrospective trial, some of the measurements are not typically assessed during the normal treatment so there had to be some planned measurments	1
4. **Endpoints appropriate to the aim of the study:** unambiguous explanation of the criteria used to evaluate the main outcome which should be in accordance with the question addressed by the study. Also, the endpoints should be assessed on an intention-to-treat basis.	
Answer: Appropriately chosen measurements to access the outcome, explained in introduction and evaluated in discussion section: “Endpoints were all cause mortality, freedom from any endoleak, target vessel patency, and re-intervention”	2
5. **Unbiased assessment of the study endpoint**: blind evaluation of objective endpoints and double-blind evaluation of subjective endpoints. Otherwise the reasons for not blinding should be stated	
Answer: No reported blind evaluation of accessed data	0
6. **Follow-up period appropriate to the aim of the study**: the follow-up should be sufficiently long to allow the assessment oft he main endpoint and possible adverse events	
Answer: Mean follow up was 14.4 months - sufficient	2
7. **Loss to follow up less than 5%**: all patients should be included in the follow up. Otherwise, the proportion lost to follow upshould not exceed the proportion experiencing the major endpoint	
Answer: “Follow up imaging was obtained in all patients” no loss in follow-up	2
8. **Prospective calculation of the study size:** information of the size of detectable difference of interest with a calculation of 95% confidence interval,	

according to the expected incidence of the outcome event, and information about the level for statistical significance and estimates of power when comparing the outcomes	
Answer: not reported	0
**Overall grade**	Overall score
Moderate quality	
	11

Scoring: 0 – not reported, 1 – reported but inadequately, 2 – reported adequately
Overall score grading: ≥8 - poor quality, 9 – 12 – moderate quality, 13 – 16 – good quality

Additional file 5

METHODOLOGICAL INDEX FOR NON-RANDOMIZED STUDIES (MINORS)

For: The Utility of Three-Dimensional Printing in Physician-Modified Stent-Grafts for Aortic Lesions Repair

Assessed study: Tong et al. Use of 3D Printing to Guide Creation of Fenestrations in Physician-Modified Stent- Grafts for Treatment of Thoracoabdominal Aortic Disease

**1. A clearly stated aim**: the question addressed should be precise and relevant in the light of available literature	Score (0-2)
Answer: The aim is described as: "To summarize the experience and outcomes of total endovascular repair of thoracoabdominal aortic disease using 3-dimensional (3D) printed models to guide on-site creation of fenestrations in aortic stent-grafts." It could be more directly described	1
**2. Inclusion of consecutive patients**: all patients potentially fit for inclusion (satisfying the criteria for inclusion) have been included in the study during the study period (no exclusion or details about the reasons for exclusion)	
Answer: inclusion was presented sufficiently: "The indications for stent-graft implantation in the setting of aortic dissection included a maximum dissecting aneu- rysm >50 mm, a false lumen diameter twice that of the true lumen and progressively enlarging (>10 mm/y), multiple tears or one tear >22 mm in diameter, ischemia of the lower extremity or branch arteries, and pain".	2
3. **Prospective collection of data:** data were collected according to a protocol established before the beginning of the study	
Answer: the study protocol is mentioned "The study protocol was approved by the institutional review board of Nanjing Drum Tower Hospital"	2
4. **Endpoints appropriate to the aim of the study:** unambiguous explanation of the criteria used to evaluate the main outcome which should be in accordance with the question addressed by the study. Also, the endpoints should be assessed on an intention-to-treat basis.	
Answer: It has been described in introduction section	2
5. **Unbiased assessment of the study endpoint**: blind evaluation of objective endpoints and double-blind evaluation of subjective endpoints. Otherwise the reasons for not blinding should be stated	
Answer: No blind evaluation reported	0
6. **Follow-up period appropriate to the aim of the study**: the follow-up should be sufficiently long to allow the assessment ofthe main endpoint and possible adverse events	
Answer: Follow up was 8.5 months – slightly shorter than the rest of studies	1
7. **Loss to follow up less than 5%**: all patients should be included in the follow up. Otherwise, the proportion lost to follow upshould not exceed the proportion experiencing the major endpoint	
Answer: One patient died 3%	2
8. **Prospective calculation of the study size:** information of the size of detectable difference of interest with a calculation of 95% confidence interval, according to the expected incidence of the outcome event, and information about the level for statistical significance and estimates of power when comparing the outcomes	
Answer: not reported	0
**Overall grade**	Overall score
Moderate quality	
	10

Scoring: 0 – not reported, 1 – reported but inadequately, 2 – reported adequately
Overall score grading: ≥8 - poor quality, 9 – 12 – moderate quality, 13 – 16 – good quality

Additional file 5

METHODOLOGICAL INDEX FOR NON-RANDOMIZED STUDIES (MINORS)

For: The Utility of Three-Dimensional Printing in Physician-Modified Stent-Grafts for Aortic Lesions Repair

Assessed study: Zheng et al. “3D Printing-Assisted versus Conventional Extracorporeal Fenestration Tevar for Stanford Type B Arteries Dissection with Undesirable Proximal Anchoring Zone: Efficacy Analysis”

**1. A clearly stated aim**: the question addressed should be precise and relevant in the light of available literature	Score (0-2)
Answer: The aim has been clearly stated: “ This study aims to evaluate the short-term and mid-term clinical outcomes, strengths and weaknesses of 3D printing-assisted extracorporeal fenestration TEVAR versus conventional extracorporeal fenestration TEVAR for treating TBAD patients with undesirable proximal anchoring zone”	2
**2. Inclusion of consecutive patients**: all patients potentially fit for inclusion (satisfying the criteria for inclusion) have been included in the study during the study period (no exclusion or details about the reasons for exclusion)	
Answer: Inclusion criteria were clearly defined: “The inclusion criteria were: (1) Diagnosed as type B AD based on the patient’s medical history and preoperative CTA, ac- cording to the AD classification criteria (Stanford classification); (2) Preoperative CTA indicated that the distance be- tween the intimal tear and LSA was <15 mm; (3) Preoperative CTA demonstrated that the dissection retrograde tear or hematoma had involved LSA; (4) No severe liver or kid- ney dysfunction.”	2
3. **Prospective collection of data:** data were collected according to a protocol established before the beginning of the study	
Answer: No reported established protocol, however the measured data has been pointed out.	1
4. **Endpoints appropriate to the aim of the study:** unambiguous explanation of the criteria used to evaluate the main outcome which should be in accordance with the question addressed by the study. Also, the endpoints should be assessed on an intention-to-treat basis.	
Answer The clinical outcomes measures included operative success rate, device deployment success rate (deined as successful positioning and release of the main stent graft during surgery, successful isolation of aneurysm, dissection proximal tear, etc.), intraoperative and postoperaive complication rate, secondary intervention rate, mortality rate, etc”	2
5. **Unbiased assessment of the study endpoint**: blind evaluation of objective endpoints and double-blind evaluation of subjective endpoints. Otherwise the reasons for not blinding should be stated	
Answer: No blinding has been implemented	0
6. **Follow-up period appropriate to the aim of the study**: the follow-up should be sufficiently long to allow the assessment of the main endpoint and possible adverse events	
Answer: The mean follow up was 16 months, which is sufficiently long	2
7. **Loss to follow up less than 5%**: all patients should be included in the follow up. Otherwise, the proportion lost to follow upshould not exceed the proportion experiencing the major endpoint	
Answer: No loss of patients in follow-up reported	2
8. **Prospective calculation of the study size:** information of the size of detectable difference of interest with a calculation of 95% confidence interval,	

according to the expected incidence of the outcome event, and information about the level for statistical significance and estimates of power when comparing the outcomes	
Answer: No prospective calculation of study size reported	0
Additional criteria in the case of comparative study	
9. **An adequate control group**: having a gold standard diagnostic test or therapeutic intervention recognized as the optimal intervention according to the available published data	
Answer: The control was a group of standard (conventional) PMSG	2
10. **Contemporary groups**: control and studied group should be managed during the same time period (no historical comparison)	
Answer: The groups came from the same period	2
11. **Baseline equivalence of groups**: the groups should be similar regarding the criteria other than the studied endpoints. Absence of confounding factors that could bias the interpretation of the results	
Answer: The groups did not have significant differences, it is presented in Table 1	2
12. **Adequate statistical analyses**: whether the statistics were in accordance with the type of study with calculation of confidence intervals or relative risk	
Answer: Statistical analysis was adequate "Data with normal distribution were analyzed by independent samples t- test, and data with non-normal distribution were analyzed by non-parametric test (Mann-Whitney U test). Categorical data were compared by chi-square test or Fisher exact test. Two-sided test, significance level α = 0.05."	2
**Overall grade**	Overall score
moderate quality	19/24

Scoring: 0 – not reported, 1 – reported but inadequately, 2 – reported adequately
Overall score grading for non-comparative: ≥8 - poor quality, 9 – 14 – moderate quality, 15 – 16 – good quality. For comparative: ≥14 - poor quality, 15 – 22 – moderate quality, 23 – 24 – good quality

Additional file 5

METHODOLOGICAL INDEX FOR NON-RANDOMIZED STUDIES (MINORS)

For: The Utility of Three-Dimensional Printing in Physician-Modified Stent-Grafts for Aortic Lesions Repair

Assessed study: Rynio et al. Initial Experience with Fenestrated Physician-Modified Stent Grafts Using 3D Aortic Templates

**1. A clearly stated aim**: the question addressed should be precise and relevant in the light of available literature	Score (0-2)
Answer: The aim/purpose of the study has been clearly stated: "The purpose of this study was to report the surgical results of PMEG using a 3D template in a center with no previous experience in complex endovascular aortic repairs"	2
**2. Inclusion of consecutive patients**: all patients potentially fit for inclusion (satisfying the criteria for inclusion) have been included in the study during the study period (no exclusion or details about the reasons for exclusion)	
Answer: The inclusion criteria has been clearly stated: "The inclusion criteria were juxtarenal and suprarenal aortic aneurysms, type IV thoracoabdominal aneurysms, and type IA endoleak after endovascular aortic repair. In asymptomatic patients, the diameter threshold for aneurysm repair was 5.5 cm in males and 5.0 cm in women. At aortic team meetings, all cases were discussed and rated as high risk for open surgery"	2
3. **Prospective collection of data:** data were collected according to a protocol established before the beginning of the study	
Answer: No reported protocol before reported however its interventional study and all needed data has been collected during surgery	1
4. **Endpoints appropriate to the aim of the study:** unambiguous explanation of the criteria used to evaluate the main outcome which should be in accordance with the question addressed by the study. Also, the endpoints should be assessed on an intention-to-treat basis.	
Answer: The endpoint is appropriate to the aim of the study, described in table 1 of this article	2
5. **Unbiased assessment of the study endpoint**: blind evaluation of objective endpoints and double-blind evaluation of subjective endpoints. Otherwise the reasons for not blinding should be stated	
Answer: No reported blind evaluation of endpoints	0
6. **Follow-up period appropriate to the aim of the study**: the follow-up should be sufficiently long to allow the assessment of the main endpoint and possible adverse events	
Answer: Follow up was appropriate, "the mean follow-up was 14 ± 12 months."	2
7. **Loss to follow up less than 5%**: all patients should be included in the follow up. Otherwise, the proportion lost to follow upshould not exceed the proportion experiencing the major endpoint	
Answer: 17 deaths during the follow -up, no information if excluded from follow-up	0
8. **Prospective calculation of the study size:** information of the size of detectable difference of interest with a calculation of 95% confidence interval, according to the expected incidence of the outcome event, and information about the level for statistical significance and estimates of power when comparing the outcomes	
Answer: not reported	
**Overall grade**	Overall score
Moderate quality	

	9

Scoring: 0 – not reported, 1 – reported but inadequately, 2 – reported adequately
Overall score grading: ≥8 - poor quality, 9 – 12 – moderate quality, 13 – 16 – good quality

Additional file 5

METHODOLOGICAL INDEX FOR NON-RANDOMIZED STUDIES (MINORS)

For: The Utility of Three-Dimensional Printing in Physician-Modified Stent-Grafts for Aortic Lesions Repair

Assessed study: Fu et al. Three-Dimensional Printing to Guide Fenestrated/Branched TEVAR in Triple Aortic Arch Branch Reconstruction With a Curative Effect Analysis

**1. A clearly stated aim**: the question addressed should be precise and relevant in the light of available literature	Score (0-2)
Answer: The aim has been stated: "Three-dimensional (3D) printing technology has become a leading manufacturing technique in health care and medicine, it enables the production of anatomically matched and patient-specific devices and constructs with high tunability and complexity.4 At our center, we used this technology to print anatomical models rather than medical devices, which allowed individualized and accurate positioning of the fenestrations for the aortic arch branches."	2
**2. Inclusion of consecutive patients**: all patients potentially fit for inclusion (satisfying the criteria for inclusion) have been included in the study during the study period (no exclusion or details about the reasons for exclusion)	
Answer: The inclusion criteria has been stated: "Indications for SG implantation included asymptomatic, degenerative, or traumatic aneurysms larger than 5 cm and all cystic aneurysms. In the setting of aortic arch dissection, the indications included a maximum dissecting aneurysm >50 mm, a false lumen diameter twice that of the true lumen and progressively enlarging (>10 mm/y), the pain in the chest or hoarseness of the voice, comorbid conditions, such as other ascending or descending aortic diseases, Marfan syndrome, family history of aortic dissection rupture."	2
3. **Prospective collection of data:** data were collected according to a protocol established before the beginning of the study	
Answer: The collected data must be planned in advance as these are the operation data, such as operation time, modification time which are not routinely measured	1
4. **Endpoints appropriate to the aim of the study:** unambiguous explanation of the criteria used to evaluate the main outcome which should be in accordance with the question addressed by the study. Also, the endpoints should be assessed on an intention-to-treat basis.	
Answer: The assessed data are presented in the table 2. Reported but inadequately,	1
5. **Unbiased assessment of the study endpoint**: blind evaluation of objective endpoints and double-blind evaluation of subjective endpoints. Otherwise the reasons for not blinding should be stated	
Answer: no blinding implemented and reported	0
6. **Follow-up period appropriate to the aim of the study**: the follow-up should be sufficiently long to allow the assessment of the main endpoint and possible adverse events	
Answer: Follow – up adequate (22.3 months)	2
7. **Loss to follow up less than 5%**: all patients should be included in the follow up. Otherwise, the proportion lost to follow upshould not exceed the proportion experiencing the major endpoint	
Answer: two patients died which equals 4.6% of the patients lost in follow -up	2
8. **Prospective calculation of the study size:** information of the size of detectable difference of interest with a calculation of 95% confidence interval,	

according to the expected incidence of the outcome event, and information about the level for statistical significance and estimates of power when comparing the outcomes	
Answer: not reported	0
**Overall grade**	Overall score
Moderate quality	9

Scoring: 0 – not reported, 1 – reported but inadequately, 2 – reported adequately
Overall score grading: ≥8 - poor quality, 9 – 12 – moderate quality, 13 – 16 – good quality
